# Dynamic Metabolic Profiles and Tissue-Specific Source Effects on the Metabolome of Developing Seeds of *Brassica napus*

**DOI:** 10.1371/journal.pone.0124794

**Published:** 2015-04-28

**Authors:** Helin Tan, Qingjun Xie, Xiaoe Xiang, Jianqiao Li, Suning Zheng, Xinying Xu, Haolun Guo, Wenxue Ye

**Affiliations:** 1 State Key Laboratory of Crop Genetics and Germplasm Enhancement, Nanjing Agricultural University, Nanjing, 210095, China; 2 State Key Laboratory for Conservation and Utilization of Subtropical Agrobioresources, South China Agricultural University, Guangzhou, 510642, China; 3 Animal Sciences National Teaching Demonstration Center, Nanjing Agricultural University, Nanjing, 210095, China; 4 Institute of Vegetables and Flowers, Chinese Academy of Agricultural Sciences, Supervision and Testing Center for Vegetable Quality, Ministry of Agriculture, Beijing, 100081, China; Huazhong University of Science and Technology, CHINA

## Abstract

Canola *(Brassica napus) *is one of several important oil-producing crops, and the physiological processes, enzymes, and genes involved in oil synthesis in canola seeds have been well characterized. However, relatively little is known about the dynamic metabolic changes that occur during oil accumulation in seeds, as well as the mechanistic origins of metabolic changes. To explore the metabolic changes that occur during oil accumulation, we isolated metabolites from both seed and silique wall and identified and characterized them by using gas chromatography coupled with mass spectrometry (GC-MS). The results showed that a total of 443 metabolites were identified from four developmental stages. Dozens of these metabolites were differentially expressed during seed ripening, including 20 known to be involved in seed development. To investigate the contribution of tissue-specific carbon sources to the biosynthesis of these metabolites, we examined the metabolic changes of silique walls and seeds under three treatments: leaf-detachment (Ld), phloem-peeling (Pe), and selective silique darkening (Sd). Our study demonstrated that the oil content was independent of leaf photosynthesis and phloem transport during oil accumulation, but required the metabolic influx from the silique wall. Notably, Sd treatment resulted in seed senescence, which eventually led to a severe reduction of the oil content. Sd treatment also caused a significant accumulation of fatty acids (FA), organic acids and amino acids. Furthermore, an unexpected accumulation of sugar derivatives and organic acid was observed in the Pe- and Sd-treated seeds. Consistent with this, the expression of a subset of genes involved in FA metabolism, sugar and oil storage was significantly altered in Pe and Sd treated seeds. Taken together, our studies suggest the metabolite profiles of canola seeds dynamically varied during the course of oil accumulation, which may provide a new insight into the mechanisms of the oil accumulation at the metabolite level.

## Introduction

Plant oils, mainly comprised of triacylglycerols (TAGs), are a major component of the human diet and are widely used in the chemical industry [1]. TAGs stored in oilseeds are predominantly synthesized from glycerol-3-P and fatty acids (FAs) in the endoplasmic reticulum of embryo cells [2–4]. To date, most of the enzymes involved in the *de novo* FA biosynthesis pathways have been well-characterized across many species [5, 6]. For example, acetyl-CoA carboxylase (ACCase), one of the most important rate-limiting enzymes in FA biosynthesis, catalyzes the conversion of acetyl-CoA to malonyl-CoA [2,7]. In a reaction catalyzed by FA synthase (FAS), the malonyl moiety of malonyl-CoA is transferred to an acyl-carrier protein (ACP), ultimately leading to the formation of C16:0 and C18:0 acyl-ACP. Subsequently, these intermediates are exported to the cytoplasm where they participate in TAG synthesis [2, 3, 8]. TAG synthesis is consecutively catalyzed by a defined group of enzymes, including diacylglycerol acyltransferase (DGAT), lysophosphatidylcholine acyltransferase (LPCAT) and phospholipid diacylglycerol acyltransferase (PDAT). Finally, the product TAG is stored in oil bodies surrounded by a phospholipid monolayer embedded with oleosins. The amount of oleosins determines the size of oil bodies and is directly linked to lipid content in seeds [9–12].

Based on the knowledge of the oil biosynthesis pathway and its regulatory networks, most recent studies have attempted to stimulate FA biosynthesis by manipulating expression of oil related genes. Numerous genes involved in FA synthesis and TAG assembly, as well as transcription factors participating in oil metabolism, have been investigated in several species by genetic and molecular manipulation to change the levels of lipids [13–18]. Among these genes, *WRINKLED1*, which encodes a member of the AP2/EREB transcription factor family, has been shown to control sugar and FA metabolism in plant [19–22]. *BnWRI1* in *B*. *napus* coordinates the FA biosynthesis and photosynthesis pathways to regulate oil accumulation in *B*. *napus* [17]. However, the increases in FA levels resulting from most transgenes are limited without concerning the demands of carbon sources and the partitioning of carbon among starch, oil, and protein in seeds [2, 23].

In *B*. *napus*, carbon source is mainly assimilated by leaf photosynthesis for plant growth at early developmental stages, but declined dramatically in the leaf photosynthetic area after the initiation of senescence at the reproductive growth stage [24]. Interestingly, in the absence of leaves, the silique wall, and likely the stem tissues, can serve as carbon sources for seed development during the late growth phase, which is a crucial period for oil accumulation [25–27]. Suc is the primary form of carbon and generally exported from silique (source) via the phloem into developing seeds (sink). The imported Suc may be cleaved by invertase and then converted to hexose phosphates which can enter the respiratory pathways via glycolysis to provide substrates and reduce power for storage product synthesis [28]. These substrates imported into plastids for FA biosynthesis are mainly pyruvic acids as well as some intermediates of glycolysis and tricarboxylic acid cycle (TAC) [2, 29]. A subset of these intermediates can function as precursors for amino acid synthesis, which is required for storage protein translation. Therefore, balancing the partitioning of carbon among the oil, protein and other storage compounds is critical for the developing seeds [23, 30]. However, the endogenous metabolic status of developing seeds remains to be defined, particularly the dynamic changes of metabolites that occur during oil accumulation.

A complete understanding of the dynamic changes in crucial intermediates which affect oil accumulation is required to elucidate the influence of carbon sources on metabolic dynamics during the seed maturation phase in *B*. *napus*. To this end, this study first examined the dynamic changes of metabolites to generate a global picture of metabolites present at different stages during *B*. *napus* seed development. Tissue-specific effects of carbon sources on metabolites were tested by treating plants with leaf detachment (Ld), phloem-peeling (Pe), and silique-darkening (Sd). Using GC-MS, 20 differentially expressed metabolites were identified, which are associated with oil accumulation and may be potential targets for enhancing oil content in *B*. *napus* seeds. In addition, our results indicated that the influx of metabolites into oil was dramatically altered following Pe and Sd treatments compared to untreated controls (cont). Consistently, we also found altered expression of a subset of genes involved in FA synthesis and TAG storage. Taken together, our findings may improve our understanding of the biochemical processes responsible for oil accumulation, leading to development of methods for increasing content and quality of oil in *B*. *napus* seeds.

## Materials and Methods

Plants of oilseed rape (*B*. *napus*) cultivars Zhongshuang 11 were sown in late September 2012 and grew under standard field conditions with a seedling density of 20 × 40 cm. To investigate the dynamic metabolite profile of seeds at the filling stage, the flowering days of 30 plants were tagged. At 35 days after flowering (DAF), 42 DAF, 49 DAF and 56 DAF, siliques were harvested from the plants and chilled on ice for up to 2 h prior to seed collection. Developing seeds were removed from their siliques and stored in liquid nitrogen until they were used for extracting metabolites. In order to define the role of photoassimilates in oil accumulation in both leaves and silique walls, plants were subjected to one of three treatments. First, all leaves of 30 plants were detached when plants began to flower (leaf detachment, Ld); second, a girdling treatment was performed by peeling a blade around the green phloem in 30 stems under the tagged siliques at 25 DAF (Phloem-peeled, Pe), at this time the seed oil content begins to rapidly accumulate [30]; third, the siliques of 30 plants were covered by opaque black cloth (Silique Darkening, Sd). After two weeks, all the siliques which were girdled or subjected to darkness were harvested. Silique walls and seeds were separated for analysis as described below. Six biological replicates were analyzed, where each replicate consisted of five plant samples.

### Contents of seed lipids and chlorophyll in silique walls of oilseed rape

Oil content analysis of rapeseed seeds was performed using the Soxhlet method as described previously [31]. The chlorophyll contents in the siliques walls of darkness-treated and untreated controls were determined by spectrophotometric assay as described previously [32].

### Metabolite extraction

Samples (approximately 100 mg of fresh weight) stored at -80°C were ground in liquid nitrogen and transferred to 10 mL centrifuge tubes. Pre-cooled (-40°C) pure methanol (1.4 mL) was added and then vortexed for 10 s, followed by the addition of 60 μL Ribitol (0.2 mg/mL stock in dH_2_O) as an internal quantitative standard and the mix samples were vortexed for 10 s. Tubes were placed into an ultrasound machine at 70°C for 30 min, then 1.4 mL ddH_2_O (4°C) was added and vortexed for 1 min, followed by centrifugation at 11,000 g for 10 min. Then 1 mL supernatant was transferred into a new Eppendorf tube and dried under moderate nitrogen. The dried samples were dissolved in methoxyamine pyridine (60 μL of a 15 mg/mL solution) and vortexed for 30 s, and then incubated for 90 min at 37°C. Lastly, 60 μL of MSTFA reagent (containing 1% TMCS) were added into the mixture and incubated for 60 min at 37°C.

### GC-MS analysis

The extracted samples were analyzed using an Agilent 7890A GC system coupled to an Agilent 5975C inert XL EI/CI mass spectrometric detector (MSD) system (Agilent Technologies, Santa Clara, CA, USA). Gas chromatography was performed on an HP-5MS capillary column (5% phenyl/95% methylpolysiloxane (30 m × 250 μm i.d., 0.25 μm film thickness, Agilent J & W Scientific, Folsom, CA, USA) to separate the derivatives. The injection temperature was 280°C, the interface was set to 150°C and the ion source was adjusted to 250°C. The temperature gradient program was as follows: Initial temperature of 40°C for 6 min, +10°C/min up to 300°C and a hold at 300°C for 6 min. Mass spectrometry was determined by the full-scan method ranging from 35 to 780 (m/z).

### Metabolites identification and data analysis

Raw GC/MS data were converted into CDF format (NetCDF) using Agilent GC/MS 5975 data analysis software and were subsequently processed by the XCMS (www.bioconductor.org) using XdCMS default settings with the following changes: xcmsSet (fwhm = 3, snthresh = 3, max = 300, mzdiff = 0.5, step = 0.1, steps = 2), rector (method = “linear,” family = “gaussian”, plottype = “mdevden”) and bandwidth (bw) of 5. Each metabolite was expressed as peak area normalized to the Ribitol internal standard. For multivariate statistical analysis, the XCMS output was further processed using Microsoft Excel (Microsoft, Redmond, WA, USA). Finally, normalized data were imported into Simca-P software (version 11.0, http://www.umetrics.com/simca) for multivariate statistical analyses, including principal components analysis (PCA) and partial least squares discriminant analysis (PLS-DA). All data were mean-centered and Pareto-scaled prior to PCA and PLS-DA. Discriminating metabolites were identified using a statistically significant threshold of variable influence on projection (variable influence on projection values, VIP > 1.0) values obtained from the PLS-DA model and were further validated by *t*-test analysis. Metabolites with VIP values greater than 1.0 and *P* values below 0.05 (threshold) were selected as discriminating metabolites between two classes of samples. Identification of metabolites in samples was performed by searching in two databases, firstly searched in the commercially integrated database NIST08, and then in the publicly available database GOLM.

### Analysis of gene expression by quantitative RT-PCR (qRT-PCR)

Total RNA was prepared using a Plant RNAeasy Prep Kit (Huasun China) according to the manufacturer’s instructions. Quantitative RT-PCR (qRT) analysis was performed as described previously [31]. *BnACTIN2* (GenBank NO.: AF111812.1) was used as an internal control in RT-PCR and qRT-PCR. Primer pairs used in qRT-PCR analyses are listed in S4 Table.

## Results

### Metabolic profiling the developing seeds

Oil rapidly accumulates in developing seeds of *B*. *napus* from approximately 25 days after flowering (DAF) to 60 DAF [30]. To analyze the total content of metabolite, samples collected at four time points (35, 42, 49 and 56 DAF), coinciding with the main stages of oil accumulation, were analyzed by gas chromatography coupled with mass spectrometry (GC-MS). In total, 443 putative metabolites were identified. By comparing to the putative metabolite mass spectra and the GOLM databse, we have tentatively annotated 77 metabolites (S1 Table), which were divided into six groups: amino acids, sugars, fatty acids, orgainic acids, nucleotides, and others.

**Fig 1:**
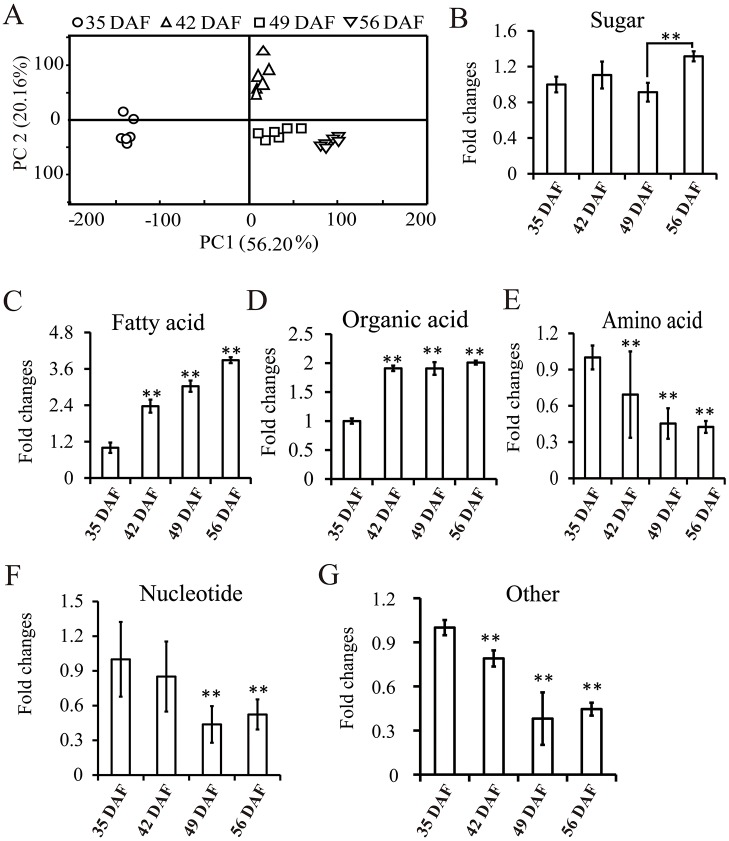
Metabolic analysis of seeds at four developmental stages. Seed samples of four developmental stages 35 days, 42 days, 49 days and 56 days after flowering were collected for metabolite analysis by GC-MS. A, PCA analysis of developing seeds from four stages; B-F, Fold changes of sugars, FA, organic acids, amino acids, nucleotides, and specialized metabolites, respectively. Data are presented as mean ± standard deviation of six replicates. DAF, days after flowering. Asterisks indicate statistically significant differences compared to control (*P < 0.05; **P < 0.01).

Principal component analysis (PCA) was used to characterize the broad patterns of changes in concentrations within 77 annotated metabolites and total metabolites, respectively. The two data sets produced similar class of separations (Fig 1A, S1 Fig). PCA modeling using the data set of 77 annotated metabolites revealed a clear and statistically significant (P < 0.05) separation of the samples into four groups, according to the time they were collected (Fig 1A). The metabolite profiles at 49 DAF and 56 DAF were more closely related than those at 35 DAF and 42 DAF, suggesting that less content of metabolites was changed after 49 DAF of seed development when the seed metabolism had reached steady state level. Further analysis indicated that the levels of sugars were increased from 35 DAF to 42 DAF and decreased from 42 DAF to 49 DAF, but dramatically increased again at 56 DAF (Fig 1B). Levels of fatty acids, which were mainly saturated fatty acids, were increased by 3-fold from 35 DAF to 56 DAF. However, the unsaturated fatty acids could not be detected in our experiment probably due to the oxidation of unsaturated fatty acids and low efficiency of MSTFA/TMCS derivatization [33, 34]. The organic acid content was increased approximately two fold from 35 DAF to 42 DAF and remained unchanged thereafter (Fig 1C and 1D). In contrast, the amino acids and nucleotide contents were decreased rapidly from 42 DAF (Fig 1E and 1F). In addition, several other metabolites, including nucleotide-associated metabolites and specialized metabolites, were significantly reduced after 42 DAF (Fig 1G).

### Identification of the altered metabolites during seed development

Among the 443 metabolites mentioned above, we identified 44 metabolites that exhibited differential changes during seed development (S2 Table). Among them, 20 metabolites and their derivatives were annotated, while the rest metabolites were unknown compounds (S2 Table). Eight out of these 20 annotated metabolites were significantly upregulated during the seed ripening (Table 1). In developing seeds, soluble sugar, including sucrose, glucose, and fructose, is the primary carbon sources for lipid synthesis. This process primarily occurred in the silique wall, followed by transporting into the developing seeds [35]. Unlike sorbose, sucrose and glucopyranose were reduced at 42 DAF, while D-mannose, raffinose and galactinol were increased at the same time point (Table 1). Sinapine is a unique metabolite in oilseeds and is the predominant phenolic compound in *B*. *napus* seeds [36]. Our results showed that the sinapine derivative cis-sinapic acid was accumulated at high levels during seed development (Table 1). All detectable organic acids were increased from 35 DAF to 42 DAF. However, their abundances were decreased between 42 DAF and 56 DAF (Table 1), implying a development-dependent change in tricarboxylic acid cycle (TCA). In contrast, we observed a dramatic decrease in amino acids, which are the major precursors of storage proteins [35], suggesting that the biosynthesis of storage proteins was completed at 35 DAF. Furthermore, we also observed some decreases in several specialized metabolites after 35 DAF (Table 1), such as epicatechin, which is the predominant flavonoid in rapeseeds. The change pattern of epicatechin is consistent with a previous report which showed the level of epicatechin was increased to its maximum level at 30 DAF, and then declined after 36 DAF [37].

**Table 1:** Differential metabolites isolated from developing seeds.

Change	Metabolites	Type	Rentions indices	VIP	35DAF	42DAF	49DAF	56DAF
Up	cis-Sinapic acid	Organic acid	2226.47	3.07	67.35 ± 14.52	1186.00 ± 367.56	1559.89 ± 276.57	1987.11 ± 107.69
	Citric acid	Organic acid	NA	3.97	2494.33 ± 267.95	4680.71 ± 422.10	3314.56 ± 319.89	3374.00 ± 140.54
	DL-malic acid	Organic acid	1479.34	4.79	2249.35 ± 80.24	3552.60 ± 201.32	5220.33 ± 605.48	5718.19 ± 197.71
	D-Mannose	carbohydrate	1815.58	4.65	1403.85 ± 41.44	2309.65 ± 191.56	1666.69 ± 158.06	3499.45 ± 83.68
	GABA	amino acid	1527.46	3.34	503.80 ± 34.30	2129.67 ± 278.40	1827.40 ± 213.09	1797.70 ± 37.00
	Galactinol	carbohydrate	2789.95	2.41	9.63 ± 3.09	8.27 ± 2.33	28.41 ± 4.93	483.79 ± 23.32
	L-Aspartic acid	amino acid	NA	1.93	772.20 ± 77.11	912.15 ± 228.40	697.85 ± 83.00	950.29 ± 42.58
	Raffinose	carbohydrate	NA	3.02	19.67 ± 11.82	248.34 ± 211.73	1023.58 ± 426.25	1276.31 ± 215.88
Down	Carbodiimide	Others	NA	1.38	377.34 ± 39.80	130.89 ± 60.20	94.30 ± 9.95	117.62 ± 5.25
	DL-Glutamine	amino acid	NA	1.85	624.85 ± 140.62	115.83 ± 46.21	89.67 ± 11.52	65.61 ± 5.06
	Epicatechin	Others	2929.72	3.58	2009.90 ± 88.51	103.03 ± 12.14	122.20 ± 92.43	37.53 ± 4.97
	Ethanolamine	Others	NA	1.36	443.28 ± 40.38	350.96 ± 135.49	235.10 ± 33.34	313.53 3± 18.24
	Glucopyranose	carbohydrate	NA	2.22	1015.83 ± 136.56	387.10 ± 172.84	332.28 ± 40.00	448.32 ± 44.80
	Glutamic acid	amino acid	NA	2.31	800.22 ± 108.20	24.63 ± 20.03	10.70 ± 4.27	19.09 ± 4.22
	L-Alanine	amino acid	1108.60	2.86	409.77 ± 150.84	847.70 ± 466.00	443.39 ± 109.29	139.24 ± 32.48
	myo-Inositol	carbohydrate	1975.96	4.37	1210.89 ± 44.23	2239.00 ± 151.77	499.24 ± 60.27	765.06 ± 57.20
	Phosphoric acid	Others	1325.19	3	2602.10 ± 212.39	2358.77 ± 189.64	1505.79 ± 252.79	1615.58 ± 74.28
	Sorbose	carbohydrate	1806.15	3.51	1848.39 ± 66.58	141.77 ± 39.73	81.84 ± 11.82	176.73 ± 14.96
	Sucrose	carbohydrate	2492.62	2.92	4742.21 ± 1100.24	5609.66 ± 1826.04	4364.78 ± 434.98	4010.97 ± 490.03
	Valine	amino acid	NA	1.1	257.84 ± 18.17	98.00 ± 48.07	67.34 ± 9.17	56.70 ± 2.66

Metabolites in seed samples of four developmental stages 35 days, 42 days, 49 days and 56 days after flowering (DAF) were analyzed by GC-MS, VIP is variable influence on projection values and NA presents that the value of retention indices has not been detected in GOLM database.

### Estimation of the effect of various tissues on oil content

**Fig 2:**
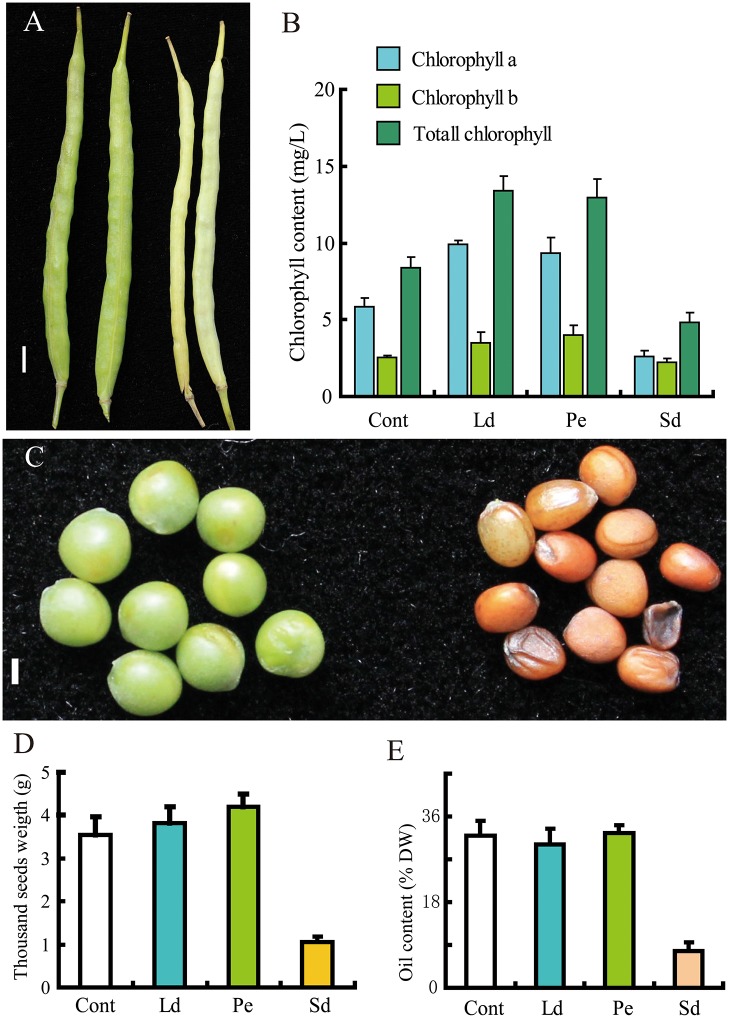
Phenotype of the seeds under different treatments. Samples were collected from the treated plants, the Ld presents the leaf was detached when plants began to flower; the Pe presents the green phloem of main branch was girdled at 25 days after flowering; the Sd presents the siliques were darkened by opaque black cloth 25 days after flowering; at 39 days after flowering, all the siliques were collected for Chlorophyll conten and GC-MS analysis. The ripened seeds were harvested for oil content anlysis. A, Silique phenotype in untreated control (Cont, right) and silique darkening treatment (Sd, left), Scale bars = 1 cm; B, Chlorophyll content of silique walls after different treatments; C, Phenotype of untreated control seed (right) and Sd-treated seeds (left), Scale bars = 1 mm; D, Oil contents of seeds (dry weight percent) after treatment: leaf detachment (Ld), phloem peeling (Pe), and silique darkening (Sd); E, Seed weight after treatment. In B, D and E, data are presented as mean ± standard deviation of six replicates.

In order to understand the role of carbon sources on metabolites changes of ripening seed, we tested the effect of leaves and siliques on oil content by three treatments, including Ld during flowering, Pe and Sd 25 days after flowering. Phenotypic analyses showed that the silique wall of Sd plants exhibited a severe albino phenotype compared to control (Fig 2A), coupling with a significant reduction of chlorophyll content in the silique wall (Fig 2B). Moreover, seeds of Sd-treated plants exhibited premature senescence, small grains, reduced weight and oil content of seed (Fig 2C–2E). In contrast, chlorophyll levels in the silique walls of Ld and Pe samples were increased compared to control (Cont) (Fig 2B). Accordingly, their respective seed weight were increased compared to that of the control (Fig 2D), however, the seed oil content was slightly increased in Pe seeds and reduced in Ld seeds (Fig 2E). These results indicate that leaf and phloem have minimal effects on oil accumulation at late developmental stages. Pe treatment induced a slight increase in seed oil content and weight, while the darkness treatment of the silique led to a dramatic loss in seed weight as a result of significant reductions of chlorophyll in the silique wall (Fig 2B and 2E). Taken together, our results suggest that the ultimate oil content of seeds was dependent on photosynthesis taking place in the silique wall but was unrelated to photosynthesis in leaf or phloem transport during oil accumulation. These findings are consistent with previously observed effects of maternal silique wall photosynthesis on oil content of *B*. *napus* seeds [38].

### Metabolites of the silique wall with different treatment

**Fig 3:**
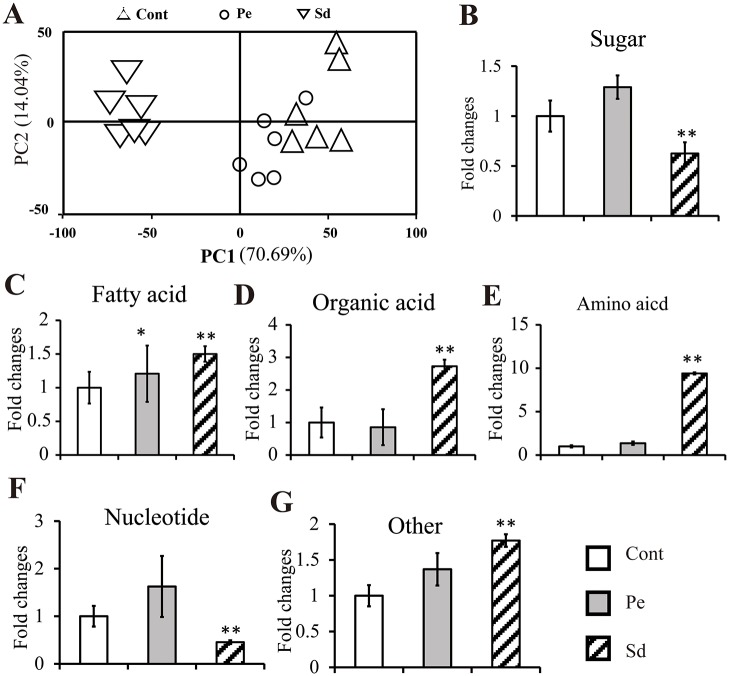
Metabolite alterations in the silique wall following Pe and Sd treatments. Silique samples were collected from the treated plants at 39 days after flowering for GC-MS analysis. A, PCA analysis of silique wall from various treatments, where Cont represents control, Pe represents phloem-peeled silique wall samples, and Sd represents silique-darkness silique wall samples; B-G, Fold changes in sugars, FA, organic acids, amino acids, nucleotides, and specialized metabolites respectively; Cont represents untreated control samples. Data are presented as mean ± standard deviation of six replicates. Asterisks indicate statistically significant differences compared to control (*P < 0.05; **P < 0.01).

During *B*. *napus* seed ripening, the silique wall plays an important role in transporting sugars into developing seeds for FA synthesis [35]. To evaluate tissue-specific metabolite changes, samples of silique walls from control (Cont), Pe and Sd plants were subjected to GC-MS analysis. Samples from Ld-treated plants were excluded due to the fact that almost no effect of this treatment occurred on seed oil content, as shown in Fig 2E. In total, 641 putative metabolites were identified and 65 metabolites were annotated in silique walls of Cont, Pe and Sd samples (S3 Table). PCA analysis using the data set of 65 annotated metabolites showed that these three treatments could be clustered into two large groups, one containing control and Pe samples and another containing Sd samples (Fig 3A). Cont and Pe samples were not distinctly separated, suggesting that blocking phloem loading has a minor effect on metabolite concentration, while silique wall photosynthesis plays a pivotal role in determining metabolite levels.

The 65 annotated metabolites were divided into six categories: amino acids, sugars, fatty acids, organic acids, nucleotides and other (specialized metabolites). The level of metabolites in the sugar and nucleotide categories were increased in Pe group but reduced in Sd group (Fig 3B and 3F). Surprisingly, we found an accumulation of FA derivatives in the Sd samples (Fig 3C), despite that a dramatic reduction of oil content was detected in these Sd samples (Fig 2E). On the other hand, there was significant variation in FA between the control and Pe samples (Fig 3C). Compared to control and Pe, the levels of organic acids, amino acids and specialized derivatives were significantly increased in Sd samples (Fig 3D, 3E and 3G), indicating that the relevant metabolite influx into seeds was largely suppressed by darkness. Consistent with that, the expression levels of sucrose transporters *BnSUC2*, *BnSUC3* and *BnSUC4* in Sd sample were lower than those in Cont and Pe (S2B Fig). No significant changes were found in amino acids and organic acid in Pe samples compared to control (Fig 3D and 3E), while nucleotides, sugars and specialized metabolites were slightly increased (Fig 3B, 3F and 3G).

Among the above 65 annotated metabolites, 32 metabolites were differentially expressed compared to control (Table 2). Interestingly, 12 out of 32 metabolites were increased in Pe and Sd samples, including organic acids and amino acid derivatives (Table 2). Only one metabolite, octadecanoic acid, was substantially decreased in both Pe and Sd samples (Table 2). Levels of additional 12 metabolites were significantly increased by Pe treatment but decreased by Sd treatment, while adenine, DL-malic acid, gluconic acid, and sucrose were increased by Sd treatment but decreased by Pe treatment (Table 2). The increased content of primary metabolites in seeds, apparently resulted from the increased influx for seed storage reserves. Our results also suggest that the transport of these metabolites was inhibited by selective darkening of the silique.

**Table 2:** Differential metabolites identified from the silique wall.

Annotation	Type	Rentions indices	VIP	Cont	Pe	Sd
Adenine	Others	NA	2.09	245.45 ± 192.04	72.93 ± 84.70	345.37 ± 199.07
Carbodiimide	Others	NA	1.65	1697.47 ± 665.39	2365.78 ± 875.93	1709.09 ± 293.75
Citric acid	Organic acid	NA	6.9	15.46 ± 4.20	21.13 ± 12.53	746.98 ± 92.61
D-Fructose	carbohydrate	1798.12	2.79	1143.71 ± 197.13	1413.96 ± 156.54	717.31 ± 169.98
D-Glucose	carbohydrate	1824.04	5.06	2307.52 ± 600.83	2764.45 ± 638.14	1752.96 ± 855.29
DL-Glutamine	amino acid	NA	1.07	958.11 ± 245.83	1138.82 ± 138.08	521.19 ± 47.06
DL-Malic acid	Organic acid	1479.34	3.34	111.01 ± 16.35	98.55 ± 9.30	505.85 ± 79.62
D-Mannose	carbohydrate	1815.58	4.33	1454.96 ± 1617.86	1035.57 ± 514.19	753.80 ± 249.01
D-Sorbose	carbohydrate	1806.15	4.75	976.03 ± 57.19	1127.59 ± 354.67	614.17 ± 200.67
D-Xylose	carbohydrate	NA	1.07	1042.03 ± 127.08	1250.12 ± 122.07	728.61 ± 127.71
Fumaric acid	Organic acid	1392.34	1.12	421.63 ± 68.50	633.73 ± 151.53	393.52 ± 31.71
GABA	amino acid	1527.46	1.92	945.04 ± 162.95	1478.95 ± 209.00	485.27 ± 80.70
Gentiobiose_1	carbohydrate	NA	1.08	362.32 ± 104.74	425.79 ± 217.86	557.86 ± 117.96
Gluconic acid	Organic acid	1907.02	1.65	5.01 ± 1.06	4.17 ± 0.68	174.79 ± 12.75
Glutamic acid	amino acid	NA	2.29	5.92 ± 0.98	7.04 ± 1.15	151.40 ± 16.77
Hexadecanoic acid	Fatty acid	2082.86	1.26	2.94 ± 2.03	3.42 ± 1.58	148.64 ± 13.34
Inositol	carbohydrate	1975.96	4.2	11.35 ± 1.75	15.71 ± 2.88	160.76 ± 47.57
Isoleucine_1	amino acid	NA	2.73	408.36 ± 64.12	510.55 ± 56.95	304.17 ± 36.38
L-Asparagine	amino acid	NA	2.83	9.04 ± 2.46	14.07 ± 4.57	147.43 ± 30.42
L-Aspartic acid	amino acid	NA	2.82	40.94 ± 21.19	119.74 ± 41.38	12.27 ± 3.55
L-Threonine	amino acid	NA	1.31	523.07 ± 117.26	617.61 ± 309.36	624.50 ± 59.63
Mannonic acid	Organic acid	NA	2.36	71.90 ± 33.56	141.89 ± 55.64	82.60 ± 11.13
Melezitose	carbohydrate	3194.35	1.37	133.62 ± 58.82	175.69 ± 158.28	92.04 ± 49.02
Octadecanoic acid	Fatty acid	NA	2.24	40.47 ± 19.25	30.05 ± 24.09	24.40 ± 8.25
P5C	Organic acid	1153.38	6.31	2249.17 ± 1128.81	2846.41 ± 644.47	1724.15 ± 337.86
Phosphoric acid	Others	1325.19	4.55	43.57 ± 15.48	61.82 ± 6.22	82.50 ± 15.63
Propanedioic acid	Organic acid	NA	1.07	135.16 ± 36.13	163.78 ± 70.98	212.67 ± 26.43
Serine_1	amino acid	NA	3.05	55.44 ± 12.22	90.44 ± 58.60	23.42 ± 0.96
Serine_2	amino acid	NA	1.86	1.145 ± 0.23	1.79 ± 0.41	74.17 ± 22.24
Sucrose	carbohydrate	2492.62	7.18	17.36 ± 9.00	14.89 ± 7.60	92.33 ± 30.05
Tyrosine	amino acid	NA	1.01	2.48 ± 0.48	4.24 ± 2.06	77.34 ± 38.44
Valine	amino acid	NA	1.4	16.42 ± 9.20	32.24 ± 20.42	25.63 ± 6.34

GC-MS analysis of metabolites in 39 days silique collected from the treated plants. The Pe presents the green phloem of main branch was girdled at 25 days after flowering. The Sd presents the siliques were darkened by opaque black cloth 25 days after flowering. Cont is the control sample without any treatment. VIP is variable influence on projection values and NA presents that the value of retention indices has not been detected in GOLM database.

### Darkness treatment reduces metabolite utilization in seeds

**Fig 4:**
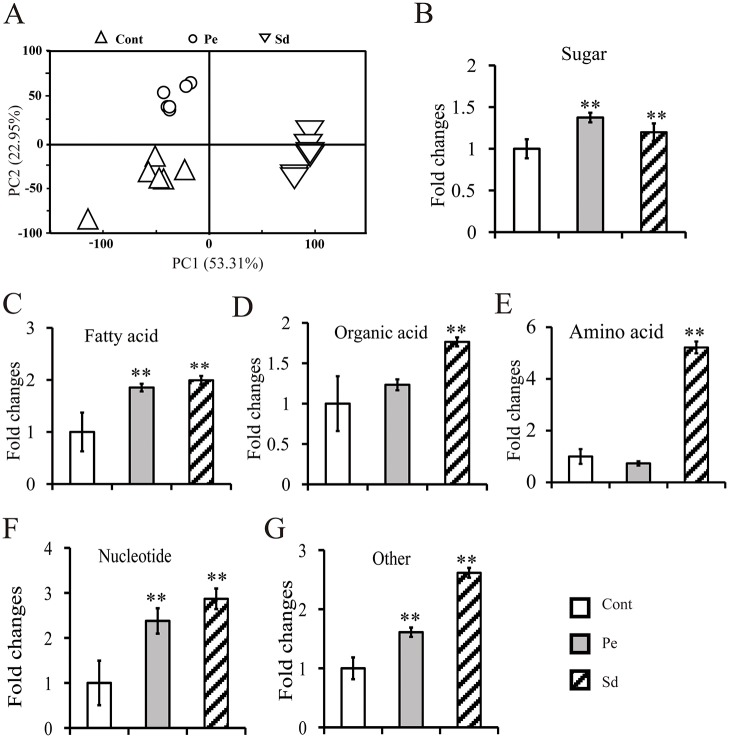
Metabolite alterations in seeds following Pe and Sd treatments. Seed samples were collected from the treated plants 39 days after flowering for GC-MS analysis. A, PCA analysis of seeds after various treatments, where Cont represents control samples, Pe represents phloem-peeled silique wall samples, and Sd represents silique-darkened silique wall samples; B-G, Fold changes of sugars, FA, organic acids, amino acids, nucleotides, and specialized metabolites, respectively; Cont represents untreated control seeds. Data are presented as mean ± standard deviation of six replicates. Asterisks indicate statistically significant differences compared to control (*P < 0.05; **P < 0.01).

To confirm the effects of darkness treatment on the metabolite content in seeds, we harvested seeds from control (Cont), Pe and Sd plants for GC-MS analysis. In total, 444 putative metabolites were identified and 78 metabolites were annotated in the seeds of control, Pe and Sd samples. PCA analysis of 78 annotated metabolites indicated that the control, Pe and Sd samples were clustered into separate groups (Fig 4A). Among the 78 annotated metabolites, we observed an accumulation of sugars in the Pe samples, as well as in the Sd samples (Fig 4B). Interestingly, the patterns of change in sugar levels were opposite between silique walls and seeds (Fig 3B and Fig 4B). Consistent with this finding, fatty acids, organic acids and amino acids, as well as specialized derivatives, were significantly increased in Sd samples (Fig 4C–4G), further indicating that darkness treatment affected metabolite influx during oil accumulation in seeds. Surprisingly, the level of FA was significantly increased in Pe seeds (Fig 4C), despite that only a slight increase was found in seed oil content (Fig 2E). These results suggest that the metabolism of FA into oil contents was inhibited in Sd seeds.

Among the 444 total metabolites, 37 metabolites exhibited differential accumulation, and only 18 metabolites could be annotated by KEGG (Table 3). Except the L-aspartic acid and 4-amino-butanoic acid (GABA), the remaining 16 metabolites, including sugars, FA and organic acids, as well as amino acids and specialized metabolites, were upregulated in Pe seeds (Table 3). In the Sd samples, cis-Sinapic acid and sorbose were significantly reduced, whereas others metabolites showed a pattern of change similar to that observed in Pe seeds (Table 3). Taken together, our results suggested that the FA biosynthesis pathway was inhibited by darkness treatment.

**Table 3:** KEGG annotated metabolites identified from treated seeds.

Annotation	Type	Rentions indices	VIP	Cont.	Pe	Sd
4-amino-Butanoic acid	amino acid	1527.46	1.48	10.28 ± 4.12	7.07 ± 0.70	194.39 ± 147.03
Carbodiimide	Others	NA	1.38	43.39 ± 23.46	133.12 ± 60.76	290.68 ± 78.96
cis-Sinapic acid	Organic acid	2226.47	1.63	96.32 ± 70.13	214.33 ± 37.95	56.20 ± 10.79
Citric acid	Organic acid	NA	2.9	3234.34 ± 1390.42	3984.61 ± 412.84	4906.01 ± 323.49
DL-malic acid	Organic acid	1479.34	4.28	1078.93 ± 118.74	1320.78 ± 155.14	2607.30 ± 317.75
D-Mannose	carbohydrate	1815.58	3.94	950.34 ± 69.96	1762.76 ± 67.46	982.86 ± 76.64
Epicatechin	Others	2929.72	3.06	245.22 ± 166.40	979.71 ± 72.88	522.19 ± 91.90
Galactose	carbohydrate	NA	4.99	4144.68 ± 327.35	5624.89 ± 251.17	4324.61 ± 297.80
Glucopyranose	carbohydrate	NA	2.41	1334.60 ± 389.74	1505.88 ± 180.31	940.31 ± 399.70
Hexadecanoic acid	Fatty acid	2082.86	1.06	103.58 ± 27.21	185.28 ± 25.81	266.77 ± 25.35
L-Alanine	amino acid	1108.6	2.51	6.50 ± 2.48	60.46 ± 37.84	631.71 ± 458.60
L-Aspartic acid	amino acid	NA	4.08	193.61 ± 129.72	21.33 ± 20.66	1084.81 ± 312.51
myo-Inositol	carbohydrate	1975.96	2.87	325.36 ± 50.60	815.94 ± 126.88	1418.35 ± 188.30
Octadecanoic acid	Fatty acid	NA	1.02	73.00 ± 31.17	157.03 ± 23.76	231.00 ± 18.23
Phosphoric acid	Others	1325.19	5.57	1887.67 ± 584.68	2066.94 ± 140.32	4245.42 ± 391.99
Raffinose	carbohydrate	NA	1.56	7.167 ± 13.75	359.68 ± 551.65	97.96 ± 19.02
Sorbose	carbohydrate	1806.15	1.09	170.49 ± 36.51	313.54 ± 25.10	379.74 ± 49.97
Sucrose	carbohydrate	2492.62	2.96	4289.98 ± 1613.36	5398.06 ± 475.40	6277.84 ± 736.58

GC-MS analysis of metabolites in 39 days seeds collected from the treated plants. The Pe presents the green phloem of main branch was girdled at 25 days after flowering. The Sd presents the siliques were darkened by opaque black cloth 25 days after flowering. Cont is the control sample without any treatment. VIP is variable influence on projection values and NA presents that the value of retention indices h has not been detected in GOLM database.

### Expression of the genes associated with oil accumulation

**Fig 5:**
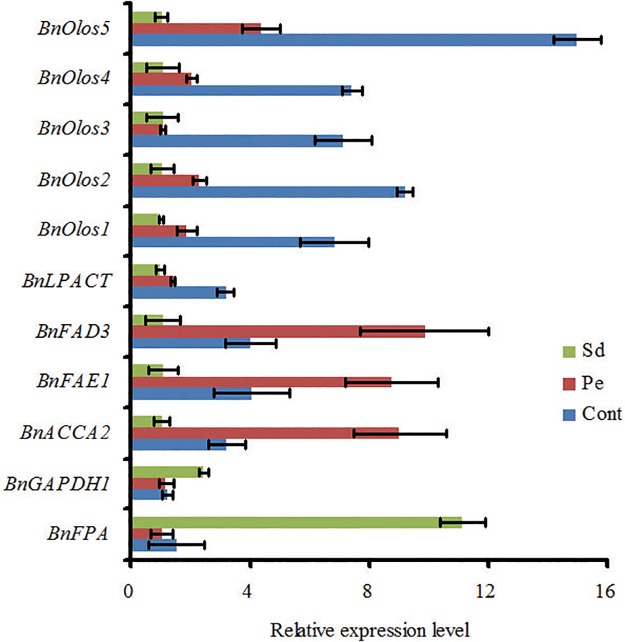
Effect of Pe and Sd treatment on expression pattern of genes involved in FA and oil contents in seeds. Seed samples were collected from the treated plants 39 days after flowering to extract total RNA for expression analysis of oil biosynthesis pathway genes. Relative expressions of related genes in TAG assembling, storage pathway, fatty acids modification and glycolytic pathway were analyzed by qRT-PCR using total RNA prepared from developing seeds with phloem peeling (Pe) and silique darkening (Sd) treatments compared to untreated control (CK). The relative expression of each gene was normalized to the *BnACTIN2* internal control. Data are presented as mean value ± standard deviation of three experiments.

Changes in metabolites during seed development are associated with changes in gene expression in these metabolic pathways. To test the effects of Pe and Sd treatments on the expression of metabolite associated genes, we performed quantitative RT-PCR for analysis of relevant transcripts involved in the biosynthesis of sugars, FA metabolism, and oil deposition in Pe and Sd-treated plants. *BnWRI1* is known to coordinate the FA biosynthesis and photosynthesis pathways to regulate oil accumulation in *B*. *napus*, and thus overexpression of *BnWRI1* results in increased chlorophyll content and biomass of seeds [17]. The expression of *BnWRI1* was dramatically upregulated in the Pe and Sd samples (S2 Fig). Consistently, the expression levels of the *BnWRI1* downstream glycolysis genes glyceraldehyde-3-phosphate dehydrogenase (*BnGAPDH1*) and glyceraldehyde-3-P dehydrogenase (*BnFPA*) in Sd sample were higher than those in Cont samples, however, the expression level of these genes in Pe was not higher than in Cont (Fig 5). In contrast, the FA metabolism genes (*BnACCA2*, *BnFAD3* and *BnFAE1*, acyl-CoA carboxylase complex A subunit, omega-3 fatty acid desaturase and fatty acid elongation1) showed a reduced expression level in Sd samples than those in Pe and Cont samples, however, an increased expression level of these genes was observed in Pe samples than Cont samples (Fig 5). Moreover, the expression of *LPCAT*, a key enzyme involved in the conversion of FA to TAG [9, 39], was decreased 3- and 5-fold in Pe and Sd samples (Fig 5), respectively, suggesting that these treatments inhibited FA metabolism. These results also confirmed our hypothesis that accumulation of FA in the context of reduced oil content in seeds results from inhibition of conversion from FA to TAG derivatives in the Sd samples and their utilization in Pe, respectively. The BnOleosin (BnOlos) family is involved in the final stages of oil deposition in *B*. *napus* [40]. Our results demonstrated that *BnOlos* expressions were reduced in Sd and Pe samples compared to Cont (Fig 5). Taken together, our results suggest that the accumulation of sugars and FA in Pe samples is due to *BnWRI1* induction, and that the slight increase in oil content in Pe samples results from increased conversion of FA to TAG. In respect to Sd samples, reduced expression of oil synthesis genes leads to significant reductions in oil content.

## Discussion

Oil content is the most important economic trait in oilseed crops, and has been extensively selected as a trait for during breeding. In this study, we present the first detailed study of metabolic changes occurred during the oil accumulation stage in the seeds and silique walls of *B*. *napus* following three treatments (Ld, Pe and Sd). We identified a series of differentially expressed metabolites, including derivatives of sugars, organic acids and FA, as well as amino acids and phenolic components. The abundance of these metabolites undergoes dynamic changes and correlates with trends of oil accumulation during seeds ripening. The understanding of the patterns of changes in metabolite composition and maturation stage-dependent metabolic shifts observed in this study may shed new light on enhancement of oil content in developing seeds.

Seed development can be divided into three stages: morphogenesis, maturation, and desiccation. During these stages, various biochemical processes and relevant metabolites undergo dynamic metabolic changes [41]. Therefore, elucidating the metabolomics dynamics associated with progression to maturity could aid in the identification of previously undescribed biomarkers that may be useful for enhancing oil content in *B*. *napus*. Our time-dependent profiling of metabolite content in developing seeds revealed a rapid shift in metabolites at 35 DAF, 42 DAF, and 49 DAF; however, there were no distinct differences in metabolites from 49 DAF to 56 DAF (Fig 1A). The production of seed storage protein peaks at 40 DAF in developing seeds of *B*. *napus* [30], which was also discovered in our study that the total amino acid content was dramatically reduced after 35 DAF (Fig 1E), such as 3- and 5-fold reductions in DL-Glutamine and Glutamic acid, respectively, at 42 DAF, while L-Alanine and Valine were increased by approximately 3-fold at this time point. Beside protein biosynthesis, the differential accumulation of L-Alanine and Valine may also contribute to the oil accumulation. Carbohydrates, including glucose, fructose, and sucrose, are the main precursors for FA biosynthesis during oil accumulation in developing seeds [31, 35, 38], however, in our metabolite profile only sucrose exhibited differential accumulation, with a rapid, approximately 3-fold reductionbetween 42 and 49 DAF. If sucrose was the primary carbon source for FA biosynthesis at mature stage, the FA content is expected to be decreased dramatically after 42 DAF (Table 1), however, we found the FA level steadily rose until 56 DAF (Fig 1C). despite a slow increase in oil accumulation starting at 25 DAF that rapidly increased from 35 to 45 DAF [30]. These results implied that other molecules might also contribute to FA biosynthesis during this process. The carbohydrates, including D-mannose, raffinose, galactinol, and sorbose, were often accumulated as antibacterial agents for storage of most plant seeds. However, the contents of raffinose, D-mannose, and sorbose were reduced by 50% at 49 DAF (Table 1), which would be utilized for FA biosynthesis. Sucrose levels remained steady during the late stage (49 to 56 DAF). The differential accumulation of sucrose and FAs in ripened seeds suggests that the biosynthetic processes had entered the stationary phase, during which substantial amounts of sucrose are no longer required [30]. Correspondingly, aside from L-Arginine, the levels of various amino acids were also significantly decreased (S1 Table, Table 1). Our previous studies also showed that the sucrose content in mature seed was significantly lower than that at 35 DAF [31]. Canola oil is the major reserved energy, and is degraded via the β-oxidative pathway to generate energy for development prior to the full establishment of photosynthetic capacity in the seedling. Therefore, high levels of free fatty acids, which are a more convenient and economical energy source than sucrose, are not only used for lipid biosynthesis, but also stored in seeds in preparation for seed germination [42, 43].

In most plants, leaves are the major photosynthetic tissue. Sucrose is produced from photosynthetically active leaves, and is transported through the phloem loading mechanism to support the growth and development of non-photosynthetic tissues. However, most leaves rapidly senesce at the reproductive growth and seed filling stage. During this time, high content of carbon source was required for seed developing [35]. In *B*. *napus*, four potential tissues can act as carbon sources to enhance metabolite production for developing seeds: leaves, residual material of senescent leaves, silique wall, and green stem. However, a series of questions regarding the contributions of these four carbon sources tissues for oil accumulation during seed ripening come out [44, 45]. We observed that the impact of leaf detachment, excluding photosynthetic effect of leaves on oil accumulation, did not significantly change oil content compared to control. The phloem peeling treatment, inhibiting phloem transfer, resulted in a slight increase in oil content compare to control. These results indicate that the silique wall, not the leaves or phloem transfer, is the major photosynthetic tissue responsible for oil accumulation during seed ripening. In agreement with our results, previous studies also concluded that the photosynthesis in silique wall may contribute the most dry matter to ripening seeds, with the contents of sucrose, fructose and glucose specifically affecting the seed oil content [26, 35, 38]. Interestingly, the sucrose level in Pe was slightly lower than that in control despite the higher chlorophyll content in Pe samples, while the sucrose and oil contents in seeds were slightly increased (Tables 2 and 3, Fig 2B and 2E). Among the 32 biomarkers exhibiting differential expression in silique, 28 of them were increased above the control to varying extent, most of which were amino acids and carbohydrates, including sucrose, fructose and glucose (Table 2). Furthermore, a part of metabolites also differentially expressed in seeds were at higher levels than those in control (Table 3). These results suggest that the content of silique metabolites can induce changes in seed metabolite levels, thus influencing oil accumulation in seeds. Therefore, these differentially expressed metabolites in silique, including but not limited to sucrose, fructose and glucose, would be potential targets to enhance the oil content of *B*. *napus* seed.

Strikingly, the silique-darkening treatment abrogated photosynthesis in the silique wall, significantly reducing both the weight and oil content of seeds (Fig 2C and 2D). The seeds of plants subjected to silique-darkness treatment were shriveled and brown, due to an insufficient supply of carbon needed for seed filling and insufficient sunlight to promote seed development. In general, the majority of oil crops (e.g. soybean, rapeseed, cotton and linseed) produce green seeds during development [15, 46], implying that the light can penetrate through the silique wall to induce chlorophyll generation in seeds, which is also associated with photosynthetic organelles containing light-harvesting pigments [47]. The photosynthetic components in green seed are proposed to provide minute carbon source as well [44]. Our previous studies indicated that the expression of metabolism genes (*SUS1*, *SUS3*, and *SUC4*) in developing seeds was significantly lower than that in the silique wall [31]. Data gathered on the role of light and photosynthesis in green oilseeds indicated that *B*. *napus* embryos are photoheterotrophic and may use light reactions to produce ATP and NADPH for oil synthesis [29, 48]. In this study, these cofactors were undetectable in metabolic profiles of the silique-darkness seeds, due to their instability during detection by GC-MS. However, a robust accumulation of sugars, amino acids, and organic acids was detected in seeds, implying that the cellular metabolic activity specifically related to oil accumulation, was inhibited by darkness (Table 3), consistent with downregulation of genes related to oil synthesis (Fig 5). Consistent with this, the levels of Alanine, an amino acid that accumulates under hypoxia-induced stress [49], was nearly 100-fold higher than control (Table 3), suggesting that the seeds developing in silique-darkness was subjected to anoxic conditions. Therefore, the supply of O_2_ may limit metabolism in developing seeds of *B*. *napus* [30]. It has been reported that the photosynthesis occurring in *B*. *napus* silique walls increases the O_2_ concentration in the silique locule, and the O_2_ released by the seed itself may be important in avoiding anoxia [30, 45, 50]. Therefore, seeds require sunlight to promote their ripening at late developmental stages. In the absence of sunlight, the metabolic activation of FA is reduced, concomitant with reduced expression of genes associated with oil biosynthesis (Fig 5).

We noted that the expression levels of *BnWRI1*, an important regulator for seed development, have increased more than 1,900-fold and 1,000-fold in Pe and Sd in our results, respectively (S2A Fig). These differential expression levels probably are that the expression patterns have changed in Pe, Sd and Cont by stress factors. On the one hand, the expression of *WRI1* depends on the development stage of seed. In Arobidopsis, the expression of *AtWRI1* begins from heart stage to walking-stich stage of developing seed. However, it is hard to detect its expression from curled stage to mature stage (http://www.arabidopsis.org/)[21]. Similarly, the *BnWRI1* also begins expression at 7 DAF in *B*. *napus* seeds, and rapidly decreases at 21 DAF [51]. In our case, the seed samples, collected at 39 DAF, *BnWRI1* could hardly express at this stage in normal growth seed (Cont). However, the seeds treated by Pe and Sd, would prolonged the development course, resulted in sustaining high expression of *BnWRI1*. On the other hand, previous study had shown that sucrose is an important signal molecular, which may induce *WRI1* expression [52]. In our results, the contents of sucrose in seeds of Pe and Sd are higher than that in Cont (Table 3). Therefore, high content of sucrose in seeds of Pe and Sd may induce expression of *BnWRI1*, resulted in high level accumulation of *BnWRI1* in seeds of Pe and Sd compared with that in Cont.

## Conclusion

Canola is a major oil crop. Understanding of dynamic changes of seed metabolic flux is a critical step to increase the quantity and quality of seed oil. We profiled metabolites in developing seeds that underlie oil accumulation process and identified a dozen differentially expressed metabolites associated with oil accumulation. Furthermore, we examined the relationship between source and sink in green oilseeds, suggesting that metabolites in silique play a critical role in oil accumulation, whereas the phloem plays a minor role. The tissue metabolite profiles show that high concentrations of metabolites in seeds transformed from silique wall induced a subset of genes related to FA synthesis and sugar metabolism to increase the metabolic flux, eventually leading to enhancing seed oil content. These findings may provide physiological and agronomical strategy for the improvement of oil quantity and quality in Brassica oilseed.

## Supporting Information

S1 Fig PCA analysis of 443 metabolite of four developing stages of seeds. Seed samples at four developmental stages 35 days, 42 days, 49 days and 56 days after flowering were collected for metabolite analysis by GC-MS. Raw GC/MS data were used to calculate concentrations by internal stander and identify metabolites via NIST08 and GOLM database. The normalized data were imported into Simca-P software (version 11.0, http://www.umetrics.com/simca) for principal component analysis (PCA).
(EPS)

S2 Fig Expression levels of genes involved in sugar metabolism and sucrose transport. Seed samples were collected from the treated plants 39 days after flowering to extract total RNA for expression analysis of oil biosynthesis pathway genes. Relative expressions of sucrose transport genes and *BnWRI1* were analyzed by qRT-PCR using total RNA prepared from developing seeds with phloem peeling (Pe) and silique darkening (Sd) treatments compared to untreated control (CK). The relative expression of each gene was normalized to the *BnACTIN2* internal control. Data are presented as mean value ± standard deviation of three experiments.
(EPS)

S1 Table Annotated metabolites in developing seeds. Metabolites in seed samples of four developmental stages 35 days, 42 days, 49 days and 56 days after flowering (DAF) were analyzed by GC-MS, VIP is variable influence on projection values. The NA presents that the value of retention indices has not been detected in GOLM database.
(XLSX)

S2 Table 44 identified metabolites exhibiting differential changes in developing seeds. Metabolites in seed samples of four developmental stages 35 days, 42 days, 49 days and 56 days after flowering (DAF) were analyzed by GC-MS, VIP is variable influence on projection values. The NA presents that the value of retention indices has not been detected in GOLM database.
(XLSX)

S3 Table Annotated metabolites among silique walls. GC-MS analyzed metabolites in 39 days siliques collected from the treated plants. The Pe presents the green phloem of main branch was girdled at 28 days after flowering. The Sd presents the siliques were darken by opaque black cloth 28 days after flowering. Cont is the control sample without any treatment. VIP is variable influence on projection values. The NA presents that the value of retention indices has not been detected in GOLM database.
(XLSX)

S4 Table Oligonucleotide primers. (XLSX)

## References

[1] Biermann U, Bornscheuer U, Meier MA, Metzger JO, Schafer HJ. Oils and fats as renewable raw materials in chemistry. Angewandte Chemie International Edition in English. 2011;50(17):3854–71. 10.1002/anie.201002767 21472903

[2] Ohlrogge J, Browse J. Lipid biosynthesis. The Plant cell. 1995;7(7):957–70. 7640528 10.1105/tpc.7.7.957 PMC160893

[3] Slabas AR, Fawcett T. The biochemistry and molecular biology of plant lipid biosynthesis. Plant molecular biology. 1992;19(1):169–91. 1600168 10.1007/BF00015613

[4] Voelker T, Kinney AJ. Variations in the biosynthesis of seed-storage lipids. Annual review of plant biology. 2001;52(1):335–61. 10.1146/annurev.arplant.52.1.335 11337402

[5] Beisson F, Koo AJ, Ruuska S, Schwender J, Pollard M, Thelen JJ, et al Arabidopsis genes involved in acyl lipid metabolism. A 2003 census of the candidates, a study of the distribution of expressed sequence tags in organs, and a web-based database. Plant physiology. 2003;132(2):681–97. 12805597 10.1104/pp.103.022988 PMC167007

[6] Millar AA, Smith MA, Kunst L. All fatty acids are not equal: discrimination in plant membrane lipids. Trends in plant science. 2000;5(3):95–101. 10707074 10.1016/s1360-1385(00)01566-1

[7] Roesler K, Shintani D, Savage L, Boddupalli S, Ohlrogge J. Targeting of the Arabidopsis homomeric acetyl-coenzyme A carboxylase to plastids of rapeseeds. Plant physiology. 1997;113(1):75–81. 9008389 10.1104/pp.113.1.75 PMC158117

[8] Harwood JL. Recent advances in the biosynthesis of plant fatty acids. Biochim Biophys Acta. 1996;1301(1–2):7–56. 8652653 10.1016/0005-2760(95)00242-1

[9] Napier JA, Haslam RP, Beaudoin F, Cahoon EB. Understanding and manipulating plant lipid composition: Metabolic engineering leads the way. Curr Opin Plant Biol. 2014;19C:68–75. 10.1016/j.pbi.2014.04.001 PMC4070482 24809765

[10] Ting JT, Lee K, Ratnayake C, Platt KA, Balsamo RA, Huang AH. Oleosin genes in maize kernels having diverse oil contents are constitutively expressed independent of oil contents. Planta. 1996;199(1):158–65. 8680304 10.1007/BF00196892

[11] Tzen J, Cao Y, Laurent P, Ratnayake C, Huang A. Lipids, Proteins, and Structure of Seed Oil Bodies from Diverse Species. Plant physiology. 1993;101(1):267–76. 12231682 10.1104/pp.101.1.267 PMC158673

[12] Tzen J, Lie G, Huang A. Characterization of the charged components and their topology on the surface of plant seed oil bodies. Journal of Biological Chemistry. 1992;267(22):15626–34. 1639802

[13] Jako C, Kumar A, Wei Y, Zou J, Barton DL, Giblin EM, et al Seed-specific over-expression of an Arabidopsis cDNA encoding a diacylglycerol acyltransferase enhances seed oil content and seed weight. Plant physiology. 2001;126(2):861–74. 11402213 10.1104/pp.126.2.861 PMC111175

[14] Mu J, Tan H, Zheng Q, Fu F, Liang Y, Zhang J, et al LEAFY COTYLEDON1 is a key regulator of fatty acid biosynthesis in Arabidopsis. Plant physiology. 2008;148(2):1042–54. 10.1104/pp.108.126342 18689444 PMC2556827

[15] Nesi N, Delourme R, Bregeon M, Falentin C, Renard M. Genetic and molecular approaches to improve nutritional value of *Brassica napus* L. seed. Comptes Rendus Biologies. 2008;331(10):763–71. 10.1016/j.crvi.2008.07.018 18926490

[16] Shorrosh BS, Roesler KR, Shintani D, van de Loo FJ, Ohlrogge JB. Structural analysis, plastid localization, and expression of the biotin carboxylase subunit of acetyl-coenzyme A carboxylase from tobacco. Plant physiology. 1995;108(2):805–12. 7610168 10.1104/pp.108.2.805 PMC157403

[17] Wu XL, Liu ZH, Hu ZH, Huang RZ. BnWRI1 coordinates fatty acid biosynthesis and photosynthesis pathways during oil accumulation in rapeseed. Journal of Integrative Plant Biology. 2014;56(6):582–93. 10.1111/jipb.12158 24393360

[18] Zou J, Katavic V, Giblin EM, Barton DL, MacKenzie SL, Keller WA, et al Modification of seed oil content and acyl composition in the brassicaceae by expression of a yeast sn-2 acyltransferase gene. The Plant cell. 1997;9(6):909–23. 9212466 10.1105/tpc.9.6.909 PMC156967

[19] Baud S, Mendoza MS, To A, Harscoet E, Lepiniec L, Dubreucq B. WRINKLED1 specifies the regulatory action of LEAFY COTYLEDON2 towards fatty acid metabolism during seed maturation in Arabidopsis. The Plant journal. 2007;50(5):825–38. 17419836 10.1111/j.1365-313X.2007.03092.x

[20] Baud S, Wuilleme S, To A, Rochat C, Lepiniec L. Role of WRINKLED1 in the transcriptional regulation of glycolytic and fatty acid biosynthetic genes in Arabidopsis. The Plant journal. 2009;60(6):933–47. 10.1111/j.1365-313X.2009.04011.x 19719479

[21] Maeo K, Tokuda T, Ayame A, Mitsui N, Kawai T, Tsukagoshi H, et al An AP2—type transcription factor, WRINKLED1, of Arabidopsis thaliana binds to the AW—box sequence conserved among proximal upstream regions of genes involved in fatty acid synthesis. The Plant journal. 2009;60(3):476–87. 10.1111/j.1365-313X.2009.03967.x 19594710

[22] Ruuska SA, Girke T, Benning C, Ohlrogge JB. Contrapuntal networks of gene expression during Arabidopsis seed filling. The Plant cell. 2002;14(6):1191–206. 12084821 10.1105/tpc.000877 PMC150774

[23] Ohlrogge JB, Jaworski JG. Regulation of fatty acid synthesis. Annual review of plant biology. 1997;48(1):109–36. 10.1146/annurev.arplant.48.1.109 15012259

[24] Pechan P, Morgan D. Defoliation and its effects on pod and seed development in oil seed rape (Brassica napus L.). Journal of experimental botany. 1985;36(3):458–68.

[25] Bennett EJ, Roberts JA, Wagstaff C. The role of the pod in seed development: strategies for manipulating yield. The New phytologist. 2011;190(4):838–53. 10.1111/j.1469-8137.2011.03714.x 21507003

[26] Lewis G, Thurling N. Growth, development, and yield of three oilseed Brassica species in a water-limited environment. Animal Production Science. 1994;34(1):93–103.

[27] Singal HR, Sheoran IS, Singh R. Photosynthetic carbon fixation characteristics of fruiting structures of *Brassica campestris* L. Plant physiology. 1987;83(4):1043–7. 16665321 10.1104/pp.83.4.1043 PMC1056498

[28] Hill LM, Morley-Smith ER, Rawsthorne S. Metabolism of sugars in the endosperm of developing seeds of oilseed rape. Plant physiology. 2003;131(1):228–36. 12529530 10.1104/pp.010868 PMC166802

[29] Browse J, Slack CR. Fatty-acid synthesis in plastids from maturing safflower and linseed cotyledons. Planta. 1985;166(1):74–80. 10.1007/BF00397388 24241314

[30] Vigeolas H, van Dongen JT, Waldeck P, Hühn D, Geigenberger P. Lipid storage metabolism is limited by the prevailing low oxygen concentrations within developing seeds of oilseed rape. Plant physiology. 2003;133(4):2048–60. 14645733 10.1104/pp.103.031963 PMC300756

[31] Tan H, Yang X, Zhang F, Zheng X, Qu C, Mu J, et al Enhanced seed oil production in canola by conditional expression of *Brassica napus LEAFY COTYLEDON1* and *LEC1-LIKE* in developing seeds. Plant physiology. 2011;156(3):1577–88. 10.1104/pp.111.175000 21562329 PMC3135965

[32] Fritschi F, Ray J. Soybean leaf nitrogen, chlorophyll content, and chlorophyll a/b ratio. Photosynthetica. 2007;45(1):92–8.

[33] Hsieh RJ, Kinsella JE. Oxidation of Polyunsaturated Fatty Acids: Mechanisms, Products, and Inhibition with Emphasis on Fish In: John EK, editor. Advances in Food and Nutrition Research. Volume 33: Academic Press; 1989 p. 233–341. 2697233 10.1016/s1043-4526(08)60129-1

[34] Ding WH, Chiang CC. Derivatization procedures for the detection of estrogenic chemicals by gas chromatography/mass spectrometry. Rapid Communications in Mass Spectrometry. 2003;17(1):56–63. 12478555 10.1002/rcm.819

[35] King SP, Lunn JE, Furbank RT. Carbohydrate content and enzyme metabolism in developing canola siliques. Plant physiology. 1997;114(1):153–60. 12223695 10.1104/pp.114.1.153 PMC158289

[36] Clauss K, von Roepenack-Lahaye E, Bottcher C, Roth MR, Welti R, Erban A, et al Overexpression of sinapine esterase *BnSCE3* in oilseed rape seeds triggers global changes in seed metabolism. Plant physiology. 2011;155(3):1127–45. 10.1104/pp.110.169821 21248075 PMC3046574

[37] Jiang J, Shao Y, Li A, Lu C, Zhang Y, Wang Y. Phenolic composition analysis and gene expression in developing seeds of yellow- and black-seeded *Brassica napus* . J Integr Plant Biol. 2013;55(6):537–51. 10.1111/jipb.12039 23445079

[38] Hua W, Li RJ, Zhan GM, Liu J, Li J, Wang XF, et al Maternal control of seed oil content in *Brassica napus*: the role of silique wall photosynthesis. The Plant journal. 2012;69(3):432–44. 10.1111/j.1365-313X.2011.04802.x 21954986

[39] Bates PD, Browse J. The pathway of triacylglycerol synthesis through phosphatidylcholine in Arabidopsis produces a bottleneck for the accumulation of unusual fatty acids in transgenic seeds. The Plant Journal. 2011;68(3):387–99. 10.1111/j.1365-313X.2011.04693.x 21711402

[40] Siloto RM, Findlay K, Lopez-Villalobos A, Yeung EC, Nykiforuk CL, Moloney MM. The accumulation of oleosins determines the size of seed oilbodies in Arabidopsis. The Plant cell. 2006;18(8):1961–74. 16877495 10.1105/tpc.106.041269 PMC1533971

[41] Weselake RJ, Taylor DC, Rahman MH, Shah S, Laroche A, McVetty PB, et al Increasing the flow of carbon into seed oil. Biotechnology advances. 2009;27(6):866–78. 10.1016/j.biotechadv.2009.07.001 19625012

[42] Huang L-S, Grunwald C. Lipid and fatty acid changes during germination of alfalfa seeds. Phytochemistry. 1990;29(5):1441–5.

[43] Watanabe M, Balazadeh S, Tohge T, Erban A, Giavalisco P, Kopka J, et al Comprehensive dissection of spatiotemporal metabolic shifts in primary, secondary, and lipid metabolism during developmental senescence in Arabidopsis. Plant physiology. 2013;162(3):1290–310. 10.1104/pp.113.217380 23696093 PMC3707545

[44] Eastmond P, Koláčá L, Rawsthorne S. Photosynthesis by developing embryos of oilseed rape (*Brassica napus* L.). Journal of Experimental Botany. 1996;47(11):1763–9.

[45] Ruuska SA, Schwender J, Ohlrogge JB. The capacity of green oilseeds to utilize photosynthesis to drive biosynthetic processes. Plant physiology. 2004;136(1):2700–9. 15347783 10.1104/pp.104.047977 PMC523334

[46] Yu CY. Molecular mechanism of manipulating seed coat coloration in oilseed Brassica species. J Appl Genet. 2013;54(2):135–45. 10.1007/s13353-012-0132-y 23329015

[47] Puthur JT, Shackira AM, Saradhi PP, Bartels D. Chloroembryos: a unique photosynthesis system. Journal of plant physiology. 2013;170(13):1131–8. 10.1016/j.jplph.2013.04.011 23706538

[48] Asokanthan PS, Johnson RW, Griffith M, Krol M. The photosynthetic potential of canola embryos. Physiologia Plantarum. 1997;101(2):353–60.

[49] De Sousa C, Sodek L. Alanine metabolism and alanine aminotransferase activity in soybean (*Glycine max*) during hypoxia of the root system and subsequent return to normoxia. Environmental and Experimental Botany. 2003;50(1):1–8.

[50] Porterfield DM, Kuang A, Smith PJ, Crispi ML, Musgrave ME. Oxygen-depleted zones inside reproductive structures of Brassicaceae: implications for oxygen control of seed development. Canadian Journal of Botany. 2000;77(10):1439–46. 11542918

[51] Jessica P. Studies on transcription factors involved in seed oil biosynthesis. M.Sc. Thesis, The University of Manitoba. 2010. Available: http://mspace.lib.umanitoba.ca/jspui/handle/1993/4363.

[52] Masaki T, Mitsui N, Tsukagoshi H, Nishii T, Morikami A, Nakamura K. ACTIVATOR of Spomin::LUC1/WRINKLED1 of Arabidopsis thaliana Transactivates Sugar-inducible Promoters. Plant and Cell Physiology. 2005;46(4):547–56. 15753106 10.1093/pcp/pci072

